# The influence of pre-motivational factors on behavior via motivational factors: a test of the I-Change model

**DOI:** 10.1186/s40359-019-0283-2

**Published:** 2019-02-20

**Authors:** Stefanie Kasten, Liesbeth van Osch, Math Candel, Hein de Vries

**Affiliations:** 1 Department of Health Promotion, Faculty of Health, Medicine and Life Sciences, Maastricht University, PO Box 616, 6200 Maastricht, MD Netherlands; 2 Department of Methodology and Statistics, Faculty of Health, Medicine and Life Sciences, Maastricht University, Maastricht, Netherlands; 3 CAPHRI-Care and Public Health Research Institute, Maastricht University, Maastricht, Netherlands

**Keywords:** Awareness, Motivational factors, Pre-motivational factors, Physical activity, Mediation

## Abstract

**Background:**

The I-Change Model for explaining motivational and behavioral change postulates that an awareness phase precedes the motivation phase of a person, and that effects of pre-motivational factors on behavior are partially mediated by motivational factors. This study tests this assumption with regard to physical activity.

**Methods:**

Observational longitudinal survey study (baseline, three months, six months) amongst Dutch adults (*N* = 2434). Structural equation modelling was used to investigate whether the influence of (1) knowledge, (2) cognizance, (3) cues, and (4) risk perception separately on intention and physical activity were mediated by motivational factors (i.e. attitudes, self-efficacy and social influence). Subsequently, a comprehensive model including all pre-motivational factors was estimated to test the same assumption for all pre-motivational factors simultaneously.

**Results:**

The results indicate that the associations of cognizance, risk perception and cues with behavior were fully mediated by motivational factors when tested separately. When tested simultaneously only the effect of cognizance remained. Cognizance was most strongly associated with positive attitudes β = .13, *p* < .01, self-efficacy β = .13, *p* < .01, and intention β = .14, *p* < .01. No direct link with behavior was found.

**Conclusion:**

The results suggest that pre-motivational factors are important to form a motivation; however, they do not directly influence behavior. The inclusion of factors such as risk perception and cognizance would help to get a better understanding of motivation formation and behavior.

## Background

Moderate to vigorous physical activity – such as cycling, sports, or walking – has shown to have essential health effects. Regular physical activity can reduce the risk for a number of non-communicable diseases such as cardiovascular diseases, diabetes, and several forms of cancer [1, 2]. Additionally, positive effects on mental health have been found with regard to depression and stress [3]. Organizations such as the World Health Organization (WHO) or the Dutch National Institute for Public Health and the Environment (RIVM) recommend for adults aged between 18 and 64 a minimum of 150 min moderate to vigorous physical activity per week [4, 5]. However, globally one in four adults is insufficiently physically active [5]. In the Netherlands less than half (44%) of the adult population adheres to the recommendations [6, 7].

Over the last decades increased attention has been paid to the problem of physical inactivity and it has become a focus of many public health interventions [8, 9]. Even though, more and more effort has been put into the development of interventions, their effectiveness is often small to moderate and their usage not wide spread [10–12]. Understanding the factors that might influence physical inactivity and knowledge about important determinants of sufficient physical activity are essential for the development of effective public health interventions [10, 13, 14].

Most interventions focus on enhancing motivational factors (i.e. attitudes, self-efficacy, intention, or social influences) [15–18] or post-motivational factors such as planning [19–21]. These interventions target populations that already have formed a basic awareness on the need to be physically active. Yet, if a person thinks that he or she is physically active but in reality may not meet the recommended standards, such a person may think that these interventions are not for him or her, as he or she is not aware of the actual situation. Similar situations are also conceivable for other behaviors, such as vegetable and snack consumption [22–24]. Whereas many social cognitive models acknowledge the importance of motivational factors with regard to health behavior, less explicit attention is paid to factors that may be relevant to a person’s self-awareness about his or her current behavior. Models such as the Trans theoretical model (TTM: [25, 26]), the Precaution Adoption Process Model [27], or the I-Change model [28, 29] assume that behavior change moves along stages or phases. Throughout these phases people develop from being unaware of their behavior to actual action taking to change health behavior. This means that to form a motivation or intention a person first needs to be aware of his or her (unhealthy) behavior and about what one could do to change the behavior. The I-Change model distinguishes a pre-motivational, motivational and post-motivational phase. The model postulates that four factors may be relevant for the pre-motivational phase [28, 29].

The first factor is knowledge, which in this case can be defined as the understanding of factual information regarding physical activity. Knowledge concerns information that leads to taking informed action (e.g. ‘the WHO recommends 150 minutes of physical activity per week’). While many interventions include methods to change knowledge, studies indicate that there is no or little direct effect of knowledge on behavior [14, 30]. However, previous research indicates that knowledge often influences motivation directly [31].

The second pre-motivational factor is behavioral cognizance. Behavioral cognizance concerns the level of a person’s awareness about his or her own health behavior. For instance, when a person correctly estimates his or her physical activity level and knows whether or not this meets recommendations, he or she is considered to be cognizant of his or her behavior. Being cognizant of one’s own behavior is an important step in the process of behavior change. However, in many cases people are unaware of their behavior and whether or not they meet suggested recommendations, which can hinder the development of motivation and actions to change [22, 32–34].

The third pre-motivational factor is risk perception. Within the I-Change model risk perception is defined as the perceived susceptibility to and the perceived severity of a health threat based on assumptions of the Health Belief Model [35] and Protection Motivation Theory [36]. Susceptibility refers to an individual’s perception of the chances of getting a disease (e.g. if I eat unhealthy, my risk of developing diabetes is [very small-very large]), whereas severity refers to an individual’s perception of the seriousness of the consequences of a disease (e.g. the consequences of diabetes are [not serious at all-very serious]). Numerous studies have confirmed the essential role of risk perception, which has an influence on behavior by influencing attitude, social influence, and self-efficacy [37, 38].

The final factor is called cues. Cues refer to hints or signals a person perceives within his or her environment (external) or himself or herself (internal) that trigger an action linked to the health behavior [39]. This includes life events (e.g. a close friend has a heart attack), but also environmental clues (e.g. a poster of a fitness club on a billboard). Environmental cues can enhance situational motivation which in turn can influence behavior directly [40]. However, until now cues have hardly been included in research and often fail to show a direct effect on behavior [41].

The I-Change model postulates that the effect of all four factors on behavior is mediated by motivational factors (i.e. attitude, self-efficacy, social influence, and intention). Support for this assumption has been found in preceding studies on sunscreen use for risk perceptions [38] and HIV prevention for risk perceptions and knowledge [31]. The aim of this study is to test the assumption of the I-Change model that the influence of all pre-motivational factors on intention and behavior is mediated by motivational factors in the case of physical activity. To investigate this hypothesis five different models are tested. Four models investigated the influence of cognizance, knowledge, risk perception, and cues separately on physical activity, and whether their effects were mediated by motivational factors. The last model tested whether these associations remained when all factors were included in one model as suggested by the theory [28, 29]. Results of this study may help to obtain insight into how motivation is formed. Furthermore, they add to the understanding of how people progress through the whole motivational process (i.e. from awareness to actual behavior) [10–12, 29, 42].

## Methods

### Participants and procedure

The study sample consisted of Dutch adults (≥ 18 years) representative for the Dutch population with regard to age, gender, educational level, and socio economic status. All participants were registered members of an online survey panel and were invited via e-mail to participate in the study. Participants were explained that confidentiality would be ensured, and that the study would comprise three measurements over a time span of 6 months. By activating a link in the e-mail, participants were directed to a web page where they could fill in the questionnaire.

Participants were excluded from the study when they indicated to not be able to be physically active due to any kind of physical disability.

### Questionnaire

#### Baseline T0

##### Demographics

Participants were asked at T0 to indicate their gender (1 = male, 2 = female), age, height, weight, and highest completed educational level. Educational level was categorized into 1 = ‘low’ (no education, elementary education, medium general secondary education, preparatory vocational school, or lower vocational school), 2 = ‘medium’ (higher general secondary education, preparatory academic education, or medium vocational school), and 3 = ‘high’ (higher vocational school or university level).

##### Knowledge

Knowledge was measured at T0 by an index of six items. Participants were presented with six statements such as ‘Regular physical activity can prevent health problems such as Diabetes Type 2 or cancer.’, and were asked to answer with ‘True’, ‘False’, ‘I don’t know’. Answering options were recoded into 1 = ‘Answered correctly’ and 0 = ‘Answered incorrectly/ not known’. A sum score was used for further analyses (max score = 6).

##### Cognizance

Cognizance was assessed by three items at T0. Participants were asked to what extent they agreed with statements such as ‘I am sufficiently physically active to maintain my health status’. Answering options ranged from 1 = ‘Absolutely disagree’ to 5 = ‘Absolutely agree’ (Cronbach’s α = .90).

##### Cues

To assess cues to action at T0, six items were used asking participants which situations would be cues that lead them to be sufficiently active. Situations included for example ‘Seeing oneself in the mirror’ or ‘Seeing physically active people in magazines, on TV, or on the internet.’ Answering options ranged from 1 = ‘No, definitely not.’ to 5 = ‘Yes, definitely.’ The higher the score, the higher the chance that a person would perceive things in his or her environment as cues to engage in a certain behavior.

##### Risk perception

Risk perception was measured by four items at T0. Two items concerned physical illness (i.e. cancer and diabetes) and two items concerned mental illness (i.e. depression) as outcome of physical inactivity. Participants were asked how severe they consider the illness, with answering options ranging from 1 = ‘Not severe at all’ to 5 = ‘Very severe’, and how high they think the risk is that they would develop the disease if they would be insufficient physically active with answering options ranging from 1 = ‘very small’ to 5 = ‘very big’ (Cronbach’s α = .63). Severity and susceptibility were combined in an additive function [38]. Including them separately did not lead to a better model fit, nor stronger effects on the motivational factors.

#### First follow-up measurement T1

##### Attitudes

Attitudes were assessed by 20 items at T1 and T0. Participants were asked to indicate to what extent they agreed with statements following the stem ‘If I am sufficiently physically active … ’. Ten items measured cons (negative attitudes) such as ‘It costs a lot of time’ or ‘I have muscle aches’ (Cronbach’s α = .88), another 10 items concerned pros (positive attitudes) such as ‘I feel better’ or ‘I have more energy’ (Cronbach’s α = .91). Pros and cons, denoted as attitude pro and attitude con respectively, were included separately in the analysis based on the assumption of the I-change model that a person tries to achieve decisional balance [28]. All analyses were corrected for baseline attitude.

##### Self-efficacy

Self-efficacy was assessed T1 and T0 by nine items following the stem ‘I find it difficult/easy to be sufficiently physically active if … ’. Items included a range of situations that have been perceived as important barriers with regard to physical activity such as bad weather or stress [43]. Answering options ranged from 1 = ‘Very difficult’ to 5 = ‘Very easy’ on a 5-point Likert scale (Cronbach’s α = .89). All analyses were corrected for baseline self-efficacy.

##### Social influence

Social influence was measured by eight items at T1 and T0. Items included social influence of the partner, family members, friends, and colleagues. Four items concerned norms asking participants to finish statements such as ‘Most of my family members … ’ with the answering options ranging from 1 = ‘definitely do not think that I need to be sufficiently physically active’ to 5 = ‘definitely think that I need to be sufficiently physically active’. Four items concerned modelling asking people to what extent they agreed with statements such as ‘Most of my friends are sufficiently physically active’. Answering options ranged from 1 = ‘Totally disagree’ to 5 = ‘Totally agree’. Both modelling and norm were combined into one latent factor social influence (Cronbach’s α = .68). Analyses were also tested with all items taken separately, and with modeling and norm as two separate factors, however, this did not lead to a better model fit or a significant change in results. We therefor opted for the most parsimonious option and included social influence as one factor. All analyses were corrected for baseline social influence.

#### Second follow-up measurement T2

##### Intention

Intention was measured with three items at T2 and T1. The first item asked whether, within the next three months, participants were planning to be sufficiently physically active. The answering options ranged from 1 = ‘No, definitely not’ to 5 = ‘Yes, definitely’. The second item assessed whether participants agreed with the statement that they were motivated to be sufficiently physical active over the course of the next three months. The answering options ranged from 1 = ‘No, absolutely not’ to 5 = ‘Yes, absolutely’. The third item asked participants to finish the statement ‘The chance that I will be sufficiently physically active within the next three months is … ’ 1 = ‘very small’ to 5 = ‘very big’ (Cronbach’s α = .93). Sufficiently physically active was defined as a minimum of 150 min of moderate-to-vigorous physical activity per week, as described in the Dutch norm. All analyses were corrected for intention after three months.

##### Physical activity

Physical activity was assessed with the International Physical Activity Questionnaire (IPAQ) – Short last seven days self-administration format [44]. The IPAQ assessed the frequency (days per week) and the duration (minutes per day) of walking, moderate-intensity activities and vigorous intensity activities. A score of minutes participants spent on being moderately to vigorously active per day was calculated. Outliers (total physical activity ≥16 h per day) were excluded from the analyses according to guidelines of the IPAQ [45]. Physical activity was measured at all three points of measurement and all analyses were corrected for baseline physical activity.

### Statistical analyses

We analyzed attrition using logistic regression, with attrition at follow-up (T2) as the outcome variable (0 = not completed; 1 = completed whole study), and age, gender, educational level, and baseline physical activity as predictors. Correlational analyses were conducted to investigate the underlying relationship between the pre-motivational factors, motivational factors and behavior. Structural Equation Modelling with MPlus Version 7.3 [46] was used to test mediation models. The model fit was estimated by the Root Mean Square Error of Approximation (RMSEA) and the comparative fit index (CFI). A good model fit is indicated by a low RMSEA (< 0.08) and a high CFI (> 0.9) [47]. Cognizance, risk perception, cues, attitudes, self-efficacy, social influence and intention were entered as latent factors. All other constructs were entered as observed variables. Five different models were investigated to test the separate effect of each pre-motivational factor (model 1–4) and the simultaneous effects (model 5).

## Results

### Attrition analysis

A total of 4978 people, representative of the Dutch adult population based on gender, age, and educational level were invited to participate in the study, of which 2434 filled in the baseline questionnaire (T0: 48,9% response rate). After 3 months 1432 participants (T1: 58,8% of baseline) filled in the questionnaire, and 1071 participants (T2: 44% of baseline) completed the questionnaire after 6 months. Logistic regression showed no differences in baseline characteristics between completers and dropouts. Based on these results and the assumptions made by the I-Change model [28] all following analyses were corrected for baseline physical activity scores, age, gender, and education level. For the structural equation modeling analyses only complete cases were used.

### Demographics

A total of 2434 people filled in the questionnaire at baseline. Of these people 364 indicated to have a chronic illness that would prevent them from being physically active, leading of a total of 2070 people. Five people were excluded as outliers due to abnormally high levels of physical activity at baseline. Of the remaining 2065 participants 47.5% were women. The mean age was 49.78 years (SD = 16.92), the majority had a medium level of education (42.8%), and participants were on average 55.89 min moderately to vigorously physically active per day (SD = 78.61).

### Correlational analyses

Table 1 shows the correlations between all pre-motivational factors at baseline, motivational factors after 3 months, and intention and behavior after 6 months. While cognizance and cues show a positive correlation with physical activity (r = .295**; r = .091** respectively), there is no significant correlation between knowledge and risk perception on the one hand, and behavior on the other. All four pre-motivational factors show significant positive correlations with intention. With regard to the other motivational factors knowledge, risk perception, and cues are most strongly correlated with attitudes pro (r = .253**, r = .339**, r = .475** respectively), while cognizance shows the strongest, but, as to be expected, negative correlation with attitudes con (r = − .496**).

**Table 1:** Correlations between pre-motivational factors, motivational factors and behavior

		Knowledge	Cognizance	Risk perception	Cues	Attitudes pro	Attitudes con	Self-efficacy	Social influence	Intention	Moderate to vigorous physical activity
Baseline (*N* = 2067)											
Knowledge	r	1	.069^b^	.211^b^	.243^b^	.253^b^	- .066^a^	,026	.152^b^	.165^b^	,009
Cognizance	r		1	.100^b^	.196^b^	.348^b^	- .496^b^	.491^b^	.128^b^	.532^b^	.295^b^
Risk perception	r			1	.315^b^	.339^b^	- .060^a^	,031	.166^b^	.205^b^	,011
Cues	r				1	.475^b^	- .132^b^	.117^b^	.212^b^	.332^b^	.091^b^
After 3 months (*N* = 1355)											
Attitudes pro	r					1	- .390^b^	.356^b^	.207^b^	.538^b^	.243^b^
Attitudes con	r						1	- .603^b^	- .149^b^	- .504^b^	- .334^b^
Self-efficacy	r							1	.123^b^	.435^b^	.350^b^
Social influence	r								1	.218^b^	,045
After 6 months (*N* = 1009)											
Intention	r									1	.334^b^
Moderate to vigorous physical activity	r										1

^a^. Correlation is significant at the 0.05 level (2-tailed)

^b^. Correlation is significant at the 0.01 level (2-tailed)

### Knowledge

**Fig. 1:**
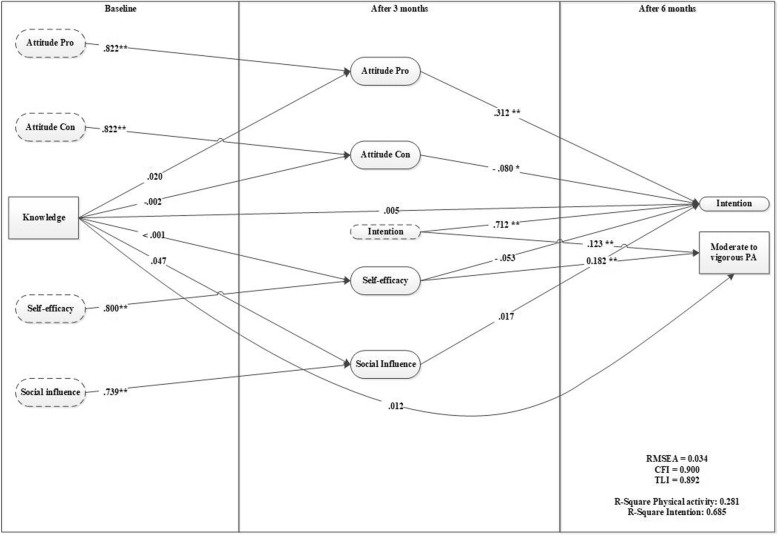
*Knowledge as a predictor for motivation and behavior (model 1)*

Model 1 (see Fig. 1) shows that knowledge has no significant effect on any of the motivational factors, intention, or physical activity. The model indicated a good model fit (RMSEA = 0.034, CFI = 0.900). The R-square for model 1 indicates that after 6 months 28.1% of variance in behavior is explained, whereas 68.5% of intention is explained.

### Cognizance

**Fig. 2:**
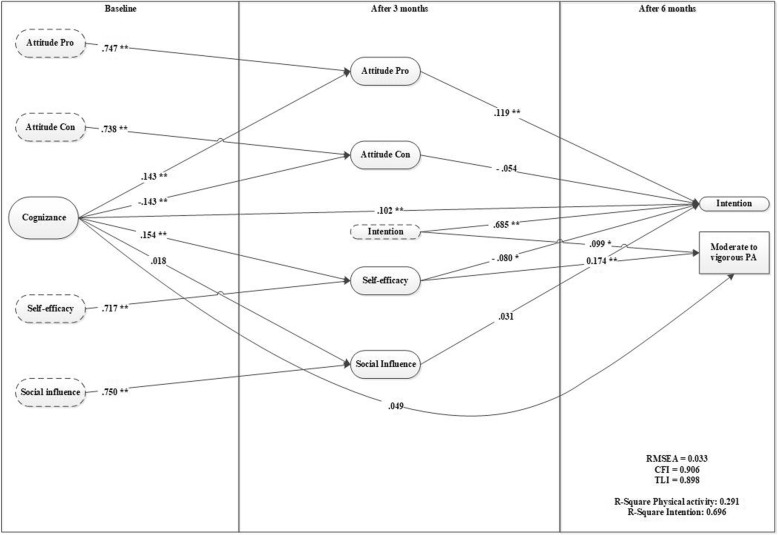
*Cognizance as a predictor for motivation and behavior (model 2)*

Model 2 (Fig. 2) indicated a good model fit (RMSEA = 0.033, CFI = 0.906). Cognizance had a strong direct predictive effect on intention but no direct effect on physical activity. The effect on behavior was fully mediated by attitudes pro, attitudes con, self-efficacy, and intention. The strongest effect of cognizance was found on self-efficacy, whereas no effect was found for social influence. This model explains 29.1% of the variance in physical activity and 69.6% of variance in intention after 6 months.

### Risk perceptions

**Fig. 3:**
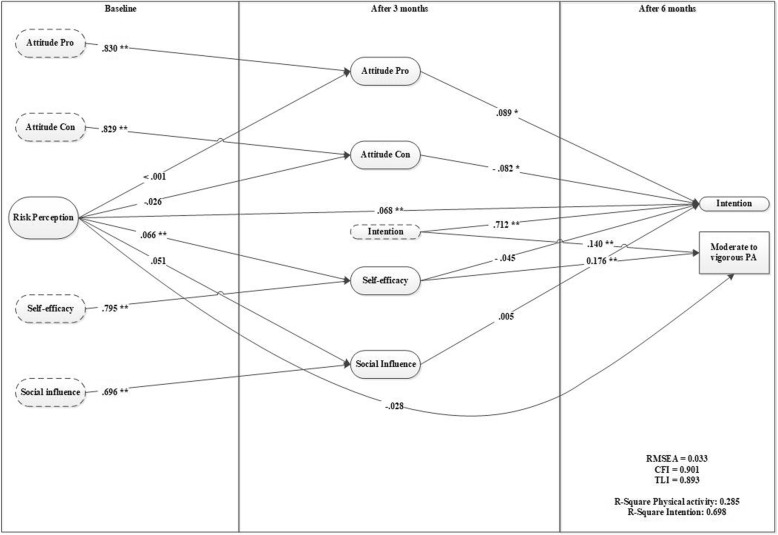
*Risk perception as a predictor for motivation and behavior (model 3)*

Model 3 (see Fig. 3) shows a direct effect of risk perception on intention, while the effect on physical activity is fully mediated by self-efficacy and intention. The model indicated a good model fit (RMSEA = 0.033, CFI = 0.901). After 6 months this model explains 28.5% of variance in physical activity and 69.8% of variance in intention.

### Cues

**Fig. 4:**
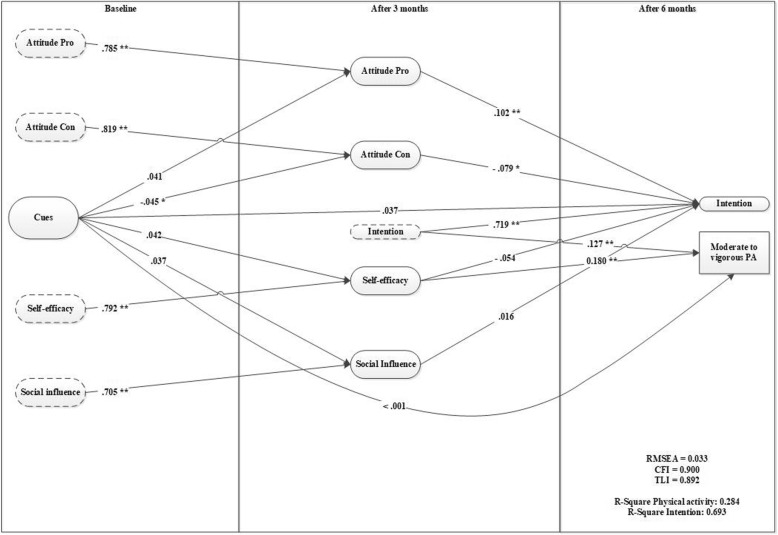
*Cues as a predictor* for motivation and behavior (model 4)

Model 4 (see Fig. 4) indicates no direct effect of cues on intention. The effect on physical activity is fully mediated by attitudes con. Model 4 explains 28.4% of the variance in physical activity and 69.3% of the variance in intention after 6 months. The model indicated a good model fit (RMSEA = 0.033, CFI = 0.900).

### Full model including all awareness factors

Finally, model 5 (Table 2) shows that when all pre-motivational factors are combined in one model, only the effects of cognizance remain. Cognizance has a direct effect on intention after 6 months, whereas its effect on behavior is mediated by attitudes, self-efficacy and intention. R-square scores indicate that the model explains 29.2% of variance in physical activity and 70.1% of the variance in intention after 6 months.

**Table 2:** Full mediation model

Dependent variable	Independent variable	Standardized regression coefficient (β)	*p*-value
Attitude Con (N3)	Attitude con (B)	0.752	0.000
	Knowledge (B)	0.009	0.658
	Cognizance (B)	−0.122	0.000
	Risk perception (B)	0.008	0.888
	Cues (B)	−0.040	0.175
Attitude Pro (N3)	Attitude Pro (B)	0.741	0.000
	Knowledge (B)	0.011	0.540
	Cognizance (B)	0.132	0.000
	Risk perception (B)	−0.020	0.620
	Cues (B)	0.036	0.184
Self-efficacy (N3)	Self-efficacy (B)	0.733	0.000
	Knowledge (B)	−0.020	0.338
	Cognizance (B)	0.132	0.000
	Risk perception (B)	0.039	0.283
	Cues (B)	0.006	0.830
Social Influence (N3)	Social Influence (B)	0.741	0.000
	Knowledge (B)	0.031	0.237
	Cognizance (B)	0.012	0.668
	Risk perception (B)	0.014	0.773
	Cues (B)	0.007	0.850
Intention (N6)	Intention (N3)	0.636	0.000
	Attitude Con (N3)	−0.074	0.056
	Attitude Pro (N3)	0.075	0.020
	Self-efficacy (N3)	−0.063	0.062
	Social Influence (N3)	0.018	0.459
	Knowledge (B)	−0.003	0.899
	Cognizance (B)	0.136	0.000
	Risk perception (B)	0.083	0.063
	Cues (B)	0.018	0.566
Behavior (N6)	Intention (N3)	0.103	0.023
	Self-efficacy (N3)	0.165	0.000
	Knowledge (B)	0.023	0.438
	Cognizance (B)	0.056	0.176
	Risk perception (B)	−0.051	0.344
	Cues (B)	0.025	0.565

B = measured at baseline, N3 = measured after three months, N6 = measured after six months

## Discussion

### Principal findings

This study aimed at investigating the hypothesis of the I-Change model that influence of pre-motivational factors with physical activity are mediated by motivational factors [28, 29]. To examine this, five different models were tested. Model one to four analyzed the separate relationship of the four proposed pre-motivational factors (i.e. knowledge, cognizance, risk perception, and cues) with behavior and motivational factors (i.e. attitudes pro, attitudes con, self-efficacy, social influences, and intention). Model five combined all four pre-motivational factors into one model. The results partially confirm the assumptions of the I-change model. While the study could not reproduce earlier findings with regard to knowledge [31], mediation effects for all other pre-motivational factors were found when looking at the separate models. However, only the mediated relationship between cognizance and behavior remained when all factors were combined in one model (model 5).

Although earlier studies showed that knowledge significantly effects motivational factors, which in turn influence behavior [14, 31], this study shows no significant association of knowledge with either motivational factors, intention or behavior. While this is not entirely in line with the assumptions of the I-Change model, the results should be considered in view of the investigated behavior. Physical activity has been promoted as an important health behavior for several decades with health agencies as well as the media endorsing the recommendations and the positive effects of physical activity on health over the past years. Variance within the level of knowledge regarding physical activity is often small [14] and as a consequence the relationship between knowledge and motivation may be weakened or even rendered insignificant.

Regarding cognizance, the results of this study indicate no significant association with physical activity, but that the relationship is fully mediated by motivational factors. This means that although awareness about one’s own behavior is not sufficient to change behavior directly, it is linked to the motivation to pursue a healthier lifestyle with regard to physical activity. These results express the importance of cognizance especially with regard to one’s attitudes and self-efficacy. Previous research shows that being aware of one’s own health behavior can be seen as a prerequisite for behavior change [22]. With regard to health behaviors such as physical activity or fruit and vegetable consumption people tend to overestimate how healthy their behavior is. This overestimation can lead to lower levels of awareness of the health risks and lower willingness to make changes [22, 48–50]. Van Sluijs, Griffin and van Poppel [23] showed that people who overestimated their physical activity were often less willing to change behavior, which shows that the misconception of one’s behavior needs to be addressed in interventions to facilitate behavior change. The sustained association between cognizance and motivation when all factors are included in the model underlines the importance of cognizance in the behavior change process and warrants further investigation. Research should focus on the level of cognizance for health behaviors, how cognizance relates to behavior change and methods to optimize cognizance.

Regarding cues, we found a weak direct association with attitude; however, no association with either intention or behavior was found. When all factors were included the relationship between cues and attitude was no longer significant. Similar to knowledge, cues are expected to be especially important when the behavior is either new or less familiar [51, 52]. As our sample was already highly active, cues might not lead to changes in motivation. This is contrary to the theoretical assumption made within the Health Belief model, which states that perceived cues have a direct effect on behavior [51]. However, earlier studies showed that perceived cues do not initiate health behavior changes directly but often led to an overall evaluation of the person’s lifestyle and situation [40, 52]. This is in line with the assumption of the I-Change model suggesting that cues can stimulate other pre-motivational factors and thus may lead to increased overall awareness; additionally, cues can lead to changes in attitudes, self-efficacy, social influences and intention [28]. As quantitative research into to the effects of cues is scarce and its operationalization varied, we recommend to investigate the effect of both internal (e.g. disease related symptoms within the individual) and external cues (e.g. external stimuli such as media exposure). Furthermore, it should be investigated whether cues would be important in later phases of behavior change such as the preparation phase [39].

Within the current literature little is known about the effect of risk perception with regard to insufficient physical activity. However, our results regarding risk perception (model 4) are in line with earlier research in other health domains. Studies with regard to healthy food consumption, sunscreen use, and condom use found no direct link between risk perception and behavior but significant association with motivational factors such as attitudes or intention [31, 38, 53]. According to the TTM, risk perception is considered a crucial factor within the pre-motivational phase [38, 54]. Within this study risk perception was associated with self-efficacy and intention contrary to findings of earlier studies that show that risk perception is mainly related to outcome expectancies [29, 38, 55]. However, this association was no longer significant when all pre-motivational factors were included into the full model. A reason could be that the studied behavior is a low risk preventive behavior for which risk perceptions might be of less importance especially when a person is already sufficiently active.

Although the results did not fully confirm the assumptions made by the I-change model and other stage models such as the TTM [25], they clearly demonstrate the importance of cognizance within the behavior change process. The results indicate that amongst a population that already is highly physically active and motivated, the pre-motivational factors knowledge, cues and risk perception do not significantly add to the prediction of behavior. However, a person’s perception of his behavior as healthy or unhealthy has a distinct contribution to the model that is not covered by the before- mentioned factors. Being aware of one’s behavior may therefore be considered as a prerequisite for motivation and behavior. While these results might indicate that we should pay more attention to cognizance, further investigation of the relationships between the pre-motivational factors and exploration of possible moderating influences of cognizance in the behavior change process is recommended. Meta-analysis concerning physical activity of Marschall and Biddle [56] showed that motivational factors such as attitude and self-efficacy are more influential in more advanced stages of behavior change. People in the earlier stages of change show less readiness to change and often perceive more barriers and lower self-efficacy [57–59]. Evidence from match-mismatch studies furthermore indicates that people who are in a pre-motivational phase benefit more from interventions that target awareness factors such as risk perception and knowledge, which would match their motivational-phases, than from interventions that target self-efficacy and attitudes which would mismatch their current motivational status [60, 61].

### Strengths and limitations

Several limitations need to be addressed when interpreting the results of this study. First, the results are based on self-reported data. Although this manner of data collection is very common, results should always be considered carefully due to the fact that participants might over –or underestimate their behavior, or respond with socially desirable answers [62, 63]. Repetition of this study or further research in this direction should make use of objective measurements such as accelerometers to ensure more reliable prediction of physical activity and to further explore accuracy of people’s performance estimations. Relatedly, little is known about the concept and operationalization of cognizance within the health domain. We currently assessed cognizance by means of the subjective perception on how healthy one’s current behavior is. However, more research is needed to investigate how we can best utilize and measure the concept within the health behavior domain, as it is conceivable that (levels of) cognizance differ substantially between various types of health behavior (e.g. physical activity vs. smoking). Third, our study assessed longitudinal associations. Intervention or manipulation of study variables took place, our results do not allow for conclusions regarding causal relationships between the different concepts. To investigate a causal relationships manipulation of the pre-motivational factors is needed. Finally, physical activity is a broad behavior that consists of many sub-behaviors. This makes it a difficult behavior to explain by one model, as, for instance an attitude towards running may differ from an attitude towards walking. For a better investigation of the mediated effect of pre-motivational factors on behavior we recommend to also test the findings for other behaviors.

Despite these limitations the study gives insight into the motivational process from pre-motivation to behavior. The study investigated all pre-motivational factors separately for physical activity for the first time and made a first attempt to investigate all four proposed pre-motivational factors of the I-change model and their effect on physical activity.

## Conclusion

The study is the first to operationalize the full I-change model to explain physical activity as a health behavior. While not supporting all assumptions of the model the study shines light on the importance of a relatively new concept with in the health domain: cognizance.

The study shows the additional contribution of cognizance and lays the basis for further investigation of pre-motivational factors.

## Abbreviations

TTM: Trans theoretical model
WHO: World health organisation
